# The first chloroplast sequence of *Rosa davurica* Pall. var. Davurica

**DOI:** 10.1080/23802359.2023.2220431

**Published:** 2023-06-13

**Authors:** Yongxiu Xia, Hua Wu, Shaofeng Li

**Affiliations:** a State Key Laboratory of Tree Genetics and Breeding, Experimental Center of Forestry in North China, National Permanent Scientific Research Base for Warm Temperate Zone Forestry of Jiulong Mountain in Beijing, Chinese Academy of Forestry, Beijing, China; b Management Center of Songshushan Nature Reserve, Inner Mongolia, China (Songshushan Forestry Center, Wengniute Banner)

**Keywords:** Rosa, *Rosa davurica* Pall, chloroplast genome, systematic evolution

## Abstract

*Rosa davurica* Pall. var. davurica is a member of the plant family Rosaceae. Although *R. davurica* has high application value, its chloroplast genome sequence has not been reported. This study aims to reveal the genetic characteristics of the chloroplast genome of *Rosa roxburghii*. The length of its total chloroplast DNA is 156,971 bp, with 37.22% G/C content. Its chloroplast genome has two inverted repeat (IRa and IRb) regions totaling 26,051 bp which are separated by a large single copy (LSC) region of 86,032 bp and a small single copy (SSC) region of 18,837 bp. The genome contains 131 independent genes (86 protein-coding, 37 tRNA, and 8 rRNA), and there are 18 repeated genes within the IR region. Among these genes, 17 genes contained one or two introns. The phylogenetic analysis showed that *R. davurica* was relatively close to other Rosa species, such as the *Rosa hybrid.*

## Introduction

The chloroplast plays an important role in plant processes, such as photosynthesis, and is a very important organ in the biosynthesis of fatty acids, starches and amino acids (Neuhaus and Emes 2000). Since the complete chloroplast genome of tobacco was first sequenced (Shinozaki et al. 1986), an increasing number of other plant chloroplast genomes have been rapidly sequenced. The ever-increasing chloroplast genome sequences provide a large foundation for studying the evolution of the plant chloroplast genome and the function and development of chloroplast genes (Shinozaki et al. 1986; Liu et al. 2012). In contrast to the nuclear genome, the chloroplast genome is relatively conserved in terms of gene composition and structure. Currently, hundreds of plant chloroplast genome sequences are available in NCBI for research.

*Rosa davurica* Pall. var. davurica (Jisaburo Ohwi 1966) is an important flowering plant in Rosaceae and is used as a good greenery and medicinal plant (Hu et al. 2011). Studies have shown that its fruits possibly contain compounds that inhibit the degranulation of rat mast cells (Kim et al. 1999). Eight known tetracyclic triterpene acids and three known flavonoids were found in *R. davurica*, which were used in traditional Chinese medicine (Kuang et al. 1989; Huo et al. 2017). *Rosa davurica* fruit and leaves could significantly prevent lipid peroxidation and inflammation (Jiao et al. 2004; Jung et al. 2011). One study found that the leaf extract of *R. davurica* had seven main components, which had a protective effect on inflammation *in vitro* and *in vivo* (HWANG et al. 2020). Studies have found that the phenolic compounds in *R. davurica* were the main biological components in its extract, which showed the strongest antioxidant effect on human low-density lipoprotein (LDL) oxidation (Sa et al. 2004). Information about the chloroplast genome sequence of *R. davurica* would help facilitate genetic research and breeding. In this paper, we report the complete chloroplast genome sequence of *R. davurica*, obtaining information for further research on the medicinal value of *R. davurica*.

## Materials and methods

**Figure 1.**
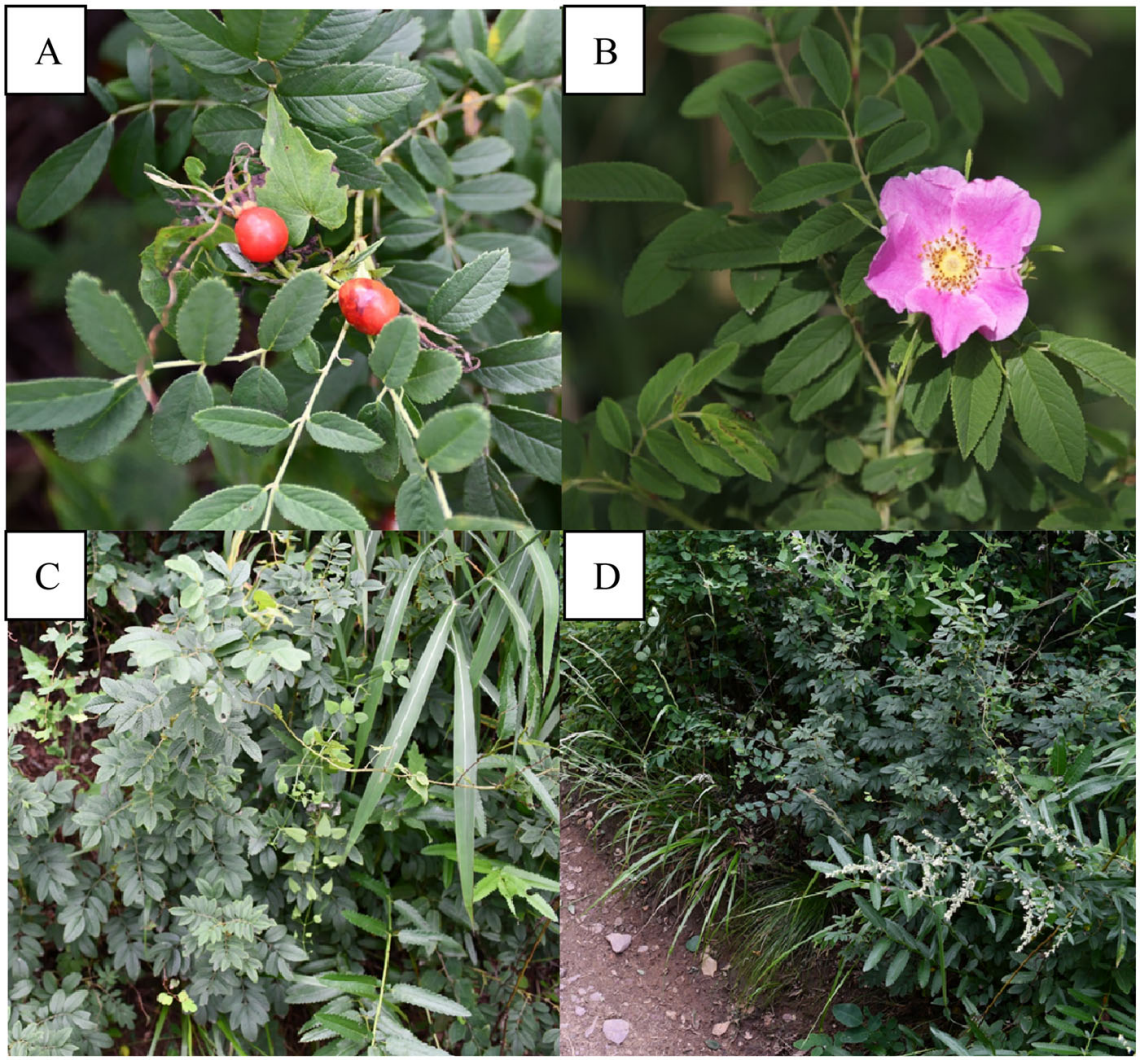
*Rosa davurica* from Jiulong Mountain in Mentougou District, Beijing, China (owned by Li et al.). (A) The fruits, orange-red, round or oval. (B) Flower, single pink, terminal. (C) Whole plant morphology. (D) Growing environment. The photos were taken by author in the Ci Mei Hua Tuo in Jiulong Mountain, Mentougou District, Beijing (coordinates: 39.944°E, 116.019°N).

Fresh *R. davurica* leaves were collected from Jiulong Mountain in Mentougou District, Beijing, China (39.944°E, 116.019°N) (Figure 1). A specimen and DNA were deposited at the herbarium of the Experimental Center of Forestry in North China (39.970°E, 116.096°N) (https://www.ncbi.nlm.nih.gov/nuccore/MW381769, contact person is Shaofeng Li and email is Lisf@caf.ac.cn) under voucher number cimei-001. The total leaf DNA was extracted by the CTAB method described by Wang (Wang et al. 2015). A TruSeq DNA sample preparation kit (Illumina, USA) was used to generate a sequencing library with a template preparation kit (Pacific Biosciences, USA). Genome sequencing was performed with the Pacific Biosciences platform and Illumina NovaSeq platform. We obtained a total of 21,586,904 reads (including 20,985,892 high-quality reads), and SPAdes (Bankevich et al. 2012) and A5-miseq (Coil et al. 2015) were performed to construct scaffolds and contigs from the clean reads. The online program Geseq (https://chlorobox.mpimp-golm.mpg.de/geseq.html) was used to upload the assembled complete chloroplast genome sequence for functional annotation. The genome of a close relative was provided for reference, and other parameters followed the default parameters. The maximum, the minimum and the average depth were 2468×, 323×, and 895.714 ×, respectively (Figure S1). The plastome map and structure of the genes (Figure S2 and S3) were drawn using the CPGview program (http://www.1kmpg.cn/cpgview/) (Liu et al. 2023). The phylogenetic analysis was performed based on the complete chloroplast genome sequence of 16 Rosa species and 2 other plants, including *P. armeniaca* and *P. salicina*. The phylogenetic relationships were determined with MAFFT using the maximum-likelihood method (Katoh et al. 2002).

**Figure 2.**
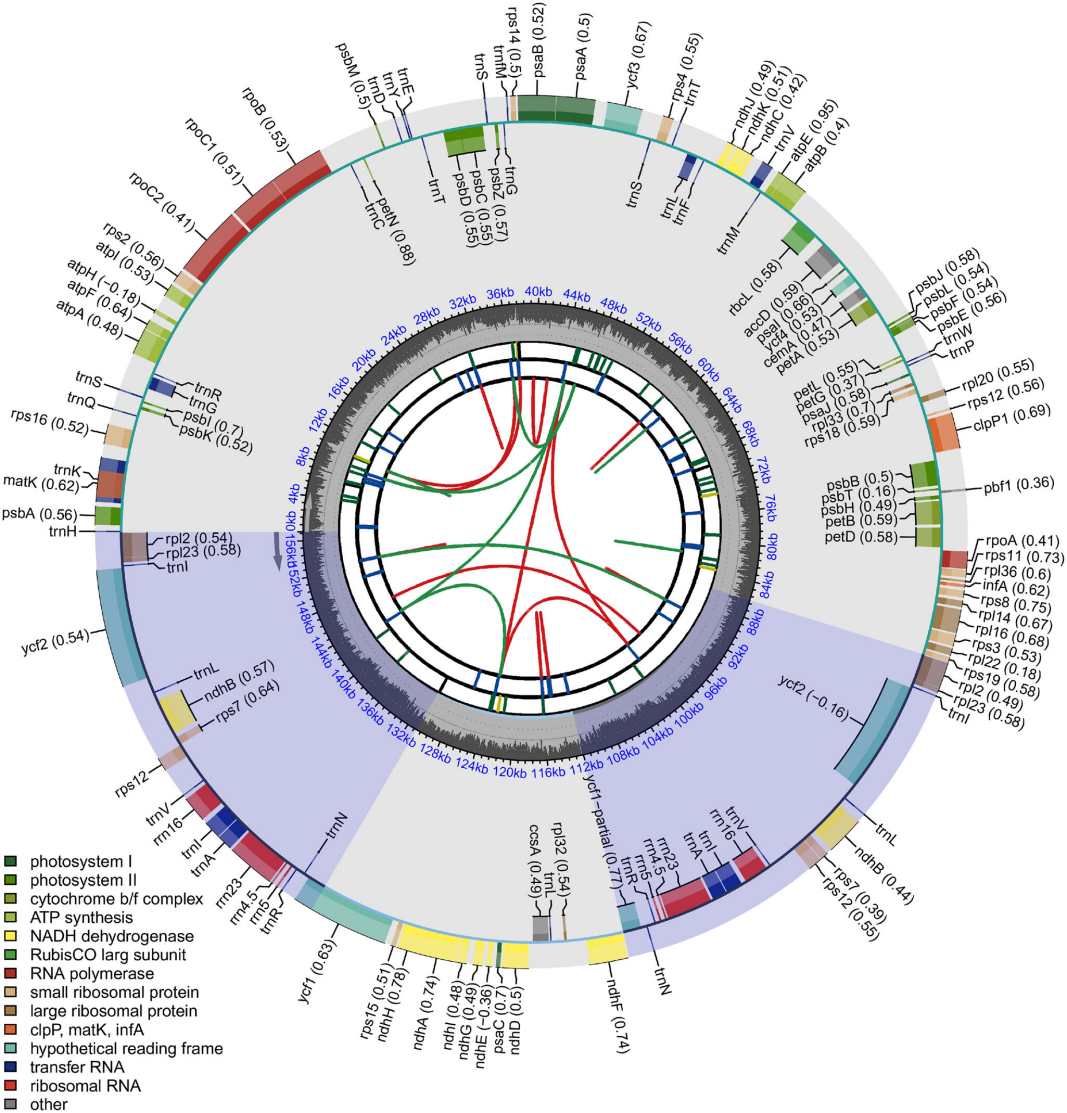
Physical map of the *Rosa davurica* whole chloroplast genome. Genes inside the circle are transcribed clockwise; genes outside are transcribed counterclockwise. The dark grey inner circle corresponds to the GC content. The chloroplast genome of *R. davurica* is 156,971 bp. The *R. davurica* chloroplast genome contains an LSC region, an SSC region and two IR regions. It encodes 131 genes, including 86 protein-coding, 8 rRNA, and 37 tRNA genes, and the genes contain one or two introns. The total GC content of the chloroplast genome was 37.22%.

**Figure 3.**
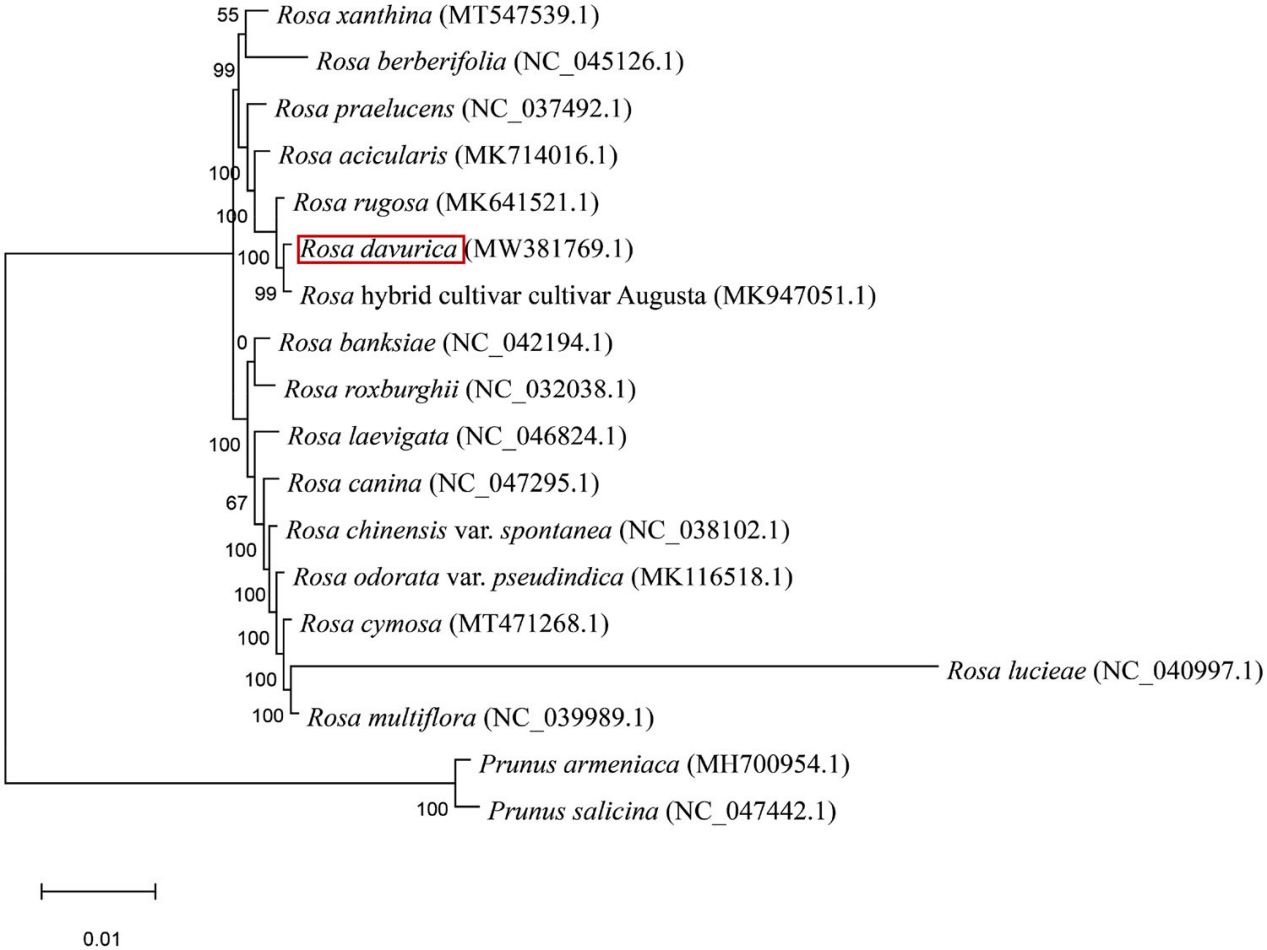
The phylogenetic tree of *R. davurica* and 17 other related species was constructed based on the complete chloroplast genome sequence with maximum likelihood, and the accession numbers are shown in the figure. GenBank accession numbers are as follows: *R. cymosa* MT471268.1 (Ding et al. 2020), *R. multiflora* NC039989.1 (Jeon and Kim 2019), *R. odorata* var*. pseudindica* MK116518.1 (Meng et al. 2019), *R. chinensis* var*. spontanea* NC038102.1 (Jian et al. 2018), *R. canina* NC047295.1 (Yin et al. 2020), *R. laevigata* NC046824.1 (Zhang et al. 2019), *R. banksiae* NC042194.1 (Wang et al. 2019), *R. roxburghii* NC032038.1 (Wang et al. 2018), *R. berberifolia* NC045126.1 (Zhang et al. 2019), *Rosa xanthina* MT547539.1 (Gao et al. 2020), *R. praelucens* NC037492.1 (Jian et al. 2018), *R. acicularis* MK714016.1(Chen et al. 2019), *R. rugosa* MK641521.1 (Jiang et al. 2019), *R. hybrid cultivar cultivar Augusta* MK947051.1 (Zhao and Gao 2020), *P. armeniaca* MH700954.1 (Xue et al. 2019), *P. salicina* NC047442.1 (Xue et al. 2019), *R. lucieae* NC040997.1 (Jeon and Kim 2019). Note: The sequence type was chloroplast complete genome; Maximum Likelihood method is adopted, and GTR is used as the alternative model. Bootstrap replicates is 1000, and bootstrap scores is the number before the branch in the diagram.

## Results

The length of the chloroplast genome sequence of *R. davurica* (accession number MW381769) is 156,971 bp, which contains an LSC region of 86,032 bp, an SSC region of 18,837 bp and an IR region of 26,051 bp. The total GC content of the chloroplast genome was 37.22% (LSC, 35.22%; SSC, 31.06%; and IR, 42.74%) (Figure 2). The chloroplast genome encodes 131 genes, including 86 protein-coding, 8 rRNA, and 37 tRNA genes. In total, 18 genes are repeated in the IR region, including 7 tRNAs, 4 rRNAs, and 7 protein-coding genes. In total, 17 genes contain introns, including 6 tRNAs, while *ycf3* and *clpP1* contain two introns. The *rps12* gene is a trans-spliced gene; the 5′-end is located in the LSC region, and the 3′-end is located in the two IR regions.

To confirm the phylogenetic position of this newly described genome, we downloaded 16 complete plastid datasets from Rosa and 2 other plants. The phylogenetic trees generated in this study also indicated a close relationship between *R. davurica* and the *R. hybrid*, with high bootstrap support (Figure 3). The chloroplast genome of *R. davurica* is a useful gene resource that could be used to improve the ecological, genetic and medicinal values of Rosaceae plants.

The complete chloroplast genome provides a wealth of genetic information and molecular markers that underlie the evolutionary relationships of land plants (Luo et al. 2014). This paper first characterized the complete chloroplast genome of *R. davurica*. The chloroplast genome of *R. davurica* is 156,971 bp (Figure 2), which provided an important basis for analyzing the evolutionary relationship of *R. davurica* in the Rosaceae plant. The *R. davurica* chloroplast genome contains an LSC region, an SSC region and two IR regions. It encodes 131 genes, including 86 protein-coding, 8 rRNA and 37 tRNA genes, and the genes contain one or two introns. The total GC content of the chloroplast genome was 37.22% (Figure 2), and these results are similar to those of other flowering plants.

## Discussion and conclusion

Chloroplast genomes have many unique advantages, including efficient transgene expression (Cosa et al. 2001). To date, transgenes have been stably integrated and expressed through the chloroplast genome to confer several useful agronomic traits, including drought tolerance (Lee et al. 2003) and salt tolerance (Kumar et al. 2004). The complete chloroplast genome sequence of *R. davurica* reported in this paper provides a basis for future research on the medicinal value and other agronomic traits of *R. davurica*.

## Supplementary Material

Supplemental Material

Supplemental Material

Supplemental Material

## Data Availability

The genome sequence data that support the findings of this study are openly available in GenBank of NCBI at [https://www.ncbi.nlm.nih.gov] (https://www.ncbi.nlm.nih.gov/) under accession no. MW381769. The associated BioProject, SRA, and Bio-Sample numbers are PRJNA701780, SRR13718192, and SAMN17910923, respectively.

## References

[CIT0001] Bankevich A, Nurk S, Antipov D, Gurevich AA, Dvorkin M, Kulikov AS, Lesin VM, Nikolenko SI, Pham S, Prjibelski AD, et al. 2012. SPAdes: a new genome assembly algorithm and its applications to single-cell sequencing. J Comput Biol. 19(5):455–477. 22506599 10.1089/cmb.2012.0021 PMC3342519

[CIT0002] Chen X, Liu Y, Sun J, Wang L, Zhou S. 2019. The complete chloroplast genome sequence of *Rosa acicularis* in Rosaceae. Mitochondrial DNA B. 4(1):1743–1744.

[CIT0003] Coil D, Jospin G, Darling AE. 2015. A5-miseq: an updated pipeline to assemble microbial genomes from Illumina MiSeq data. Bioinformatics. 31(4):587–589. 25338718 10.1093/bioinformatics/btu661

[CIT0004] Cosa BD, Moar W, Lee S-B, Miller M, Daniell H. 2001. Overexpression of the Bt Cry2Aa2 operon in chloroplasts leads to formation of insecticidal crystals. Nat Biotechnol. 19(1):71–74. 11135556 10.1038/83559 PMC4560096

[CIT0005] Ding M, Liao M, Liu P, Tan G, Chen Y, Shi S. 2020. The complete chloroplast genome of *Rosa cymosa* (Rosaceae), a traditional medicinal plant in South China. Mitochondrial DNA B Resour. 5(3):2571–2572. 33457866 10.1080/23802359.2020.1781563 PMC7782239

[CIT0006] Gao C, Wu C, Zhang Q, Wu M, Chen R, Zhao Y, Guo A, Li Z. 2020. Sequence and phylogenetic analysis of the chloroplast genome for *Rosa xanthina*. Mitochondrial DNA B Resour. 5(3):2922–2923. 33458003 10.1080/23802359.2020.1792369 PMC7782961

[CIT0007] Hu W, Han W, Jiang Y, Wang MH, Lee YM. 2011. Biological activity and inhibition of non-enzymatic glycation by methanolic extract of *Rosa davurica* Pall. roots. JFN. 16(3):242–247.

[CIT0008] Huo Y, Gao Y, Mi J, Wang X, Jiang H, Zhang H. 2017. Isolation and simultaneous quantification of nine triterpenoids from *Rosa davurica* Pall. J Chromatogr Sci. 55(2):130–136. 27733480 10.1093/chromsci/bmw155

[CIT0009] HWANG DH, Lee DY, Koh PO, Yang HR, Kang C, Kim E. 2020. *Rosa davurica* Pall. improves propionibacterium acnes-induced inflammatory responses in mouse ear edema model and suppresses pro-inflammatory chemokine production via MAPK and NF-κB pathways in HaCaT cells. IJMS. 21(5):1717. 32138302 10.3390/ijms21051717 PMC7084861

[CIT0010] Jeon JH, Kim SC. 2019. Comparative analysis of the complete chloroplast genome sequences of three closely related East-Asian wild roses (*Rosa* sect. *Synstylae*; Rosaceae). Genes. 10(1):23. 30609873 10.3390/genes10010023 PMC6356658

[CIT0011] Jian HY, Zhang YH, Yan HJ, Qiu XQ, Wang QG, Li SB, Zhang SD. 2018a. The complete chloroplast genome of a key ancestor of modern roses, *Rosa chinensis* var. *spontanea*, and a comparison with congeneric species. Molecules. 23(2):389. 29439505 10.3390/molecules23020389 PMC6017658

[CIT0012] Jian HY, Zhang SD, Zhang T, Qiu XQ, Yan HJ, Li SB, Wang QG, Tang KX. 2018b. Characterization of the complete chloroplast genome of a critically Endangered decaploid rose species, *Rosa praelucens* (Rosaceae). Conservation Genet Resour. 10(4):851–854.

[CIT0013] Jiang H, He J, Meng J. 2019. Characterization of the complete plastid genome of a Chinese Endangered Species *Rosa rugosa* Thunb. Mitochondrial Dna B. 4(1):1679–1680.

[CIT0014] Jiao SP, Chen B, Du PG. 2004. Anti-lipid peroxidation effect of *Rosa davurica* Pall. fruit. Zhong Xi Yi Jie He Xue Bao. 2(5):364–365. 15383261 10.3736/jcim20040516

[CIT0015] J P. Jisaburo Ohwi. 1966. Flora of Japan.

[CIT0016] Jung HJ, Sa JH, Song YS, Shim TH, Park EH, Lim CJ. 2011. Anti-inflammatory, anti-angiogenic, and anti-nociceptive activities of the chloroform fraction of a methanol extract from *Rosa davurica* Pall. leaves in experimental animal models. Immunopharmacol Immunotoxicol. 33(1):186–192. 20550430 10.3109/08923973.2010.491516

[CIT0017] Katoh K, Misawa K, Kuma K, Miyata T. 2002. MAFFT: a novel method for rapid multiple sequence alignment based on fast Fourier transform. Nucleic Acids Res. 30(14):3059–3066. 12136088 10.1093/nar/gkf436 PMC135756

[CIT0018] Kim HM, Park YA, Lee EJ, Shin TY. 1999. Inhibition of immediate-type allergic reaction by *Rosa davurica* Pall. in a murine model. J Ethnopharmacol. 67(1):53–60. 10616960 10.1016/s0378-8741(99)00013-6

[CIT0019] Kuang HX, Kasai R, Ohtani K, Liu ZS, Yuan CS, Tanaka O. 1989. Chemical constituents of pericarps of *Rosa davurica* Pall., a traditional Chinese medicine. Chem Pharm Bull. 37(8):2232–2233. 10.1248/cpb.37.2232 2598327

[CIT0020] Kumar S, Dhingra A, Daniell H. 2004. Plastid expressed betaine aldehyde dehydrogenase gene in carrot cultured cells, roots and leaves confers enhanced salt tolerance. Plant Physiol. 136(1):2843–2854. 15347789 10.1104/pp.104.045187 PMC523346

[CIT0021] Lee S-B, Kwon H-B, Kwon S-J, Park S-C, Jeong M-J, Han S-E, Byun M-O, Daniell H. 2003. Accumulation of trehalose within transgenic chloroplasts confers drought tolerance. Mol Breed. 11(1):1–13.

[CIT0022] Liu J, Qi ZC, Zhao YP, Fu CX, Xiang QY. 2012. Complete cpDNA genome sequence of *Smilax china* and phylogenetic placement of Liliales–influences of gene partitions and taxon sampling. Mol Phylogenet Evol. 64(3):545–562. 22643288 10.1016/j.ympev.2012.05.010

[CIT0023] Liu S, Ni Y, Li J, Zhang X, Yang H, Chen H, Liu C. 2023. CPGView: a package for visualizing detailed chloroplast genome structures. Mol Ecol Resour. 23(3):694–704. 36587992 10.1111/1755-0998.13729

[CIT0024] Luo J, Hou BW, Niu ZT, Liu W, Xue QY, Ding XY. 2014. Comparative chloroplast genomes of photosynthetic orchids: insights into evolution of the Orchidaceae and development of molecular markers for phylogenetic capplications. PLOS One. 9(6):e99016. 24911363 10.1371/journal.pone.0099016 PMC4049609

[CIT0025] Meng J, Jiang H, Zhang L, He J. 2019. Characterization of the complete plastid genome of an important Chinese Old Rose *Rosa odorata* var. *pseudindica*. Mitochondrial Dna B. 4(1):679–680.

[CIT0026] Neuhaus HE, Emes MJ. 2000. Nonphotosynthetic metabolism in plastids. Annu Rev Plant Physiol Plant Mol Biol. 51:111–140. 15012188 10.1146/annurev.arplant.51.1.111

[CIT0027] Sa JH, Lee W, Shin IC, Jeong KJ, Choi DS. 2004. Antioxidant effect of *Rosa davurica* Pall. extract on oxidation of human low density lipoprotein (LDL). Korean J Food Sci Technol. 36(2):311–316.

[CIT0028] Shinozaki K, Ohme M, Tanaka M, Wakasugi T, Hayashida N, Matsubayashi T, Zaita N, Chunwongse J, Obokata J, Yamaguchi-Shinozaki K, et al. 1986. The complete nucleotide sequence of the tobacco chloroplast genome: its gene organization and expression. Embo J. 5(9):2043–2049. 16453699 10.1002/j.1460-2075.1986.tb04464.x PMC1167080

[CIT0029] Wang H, Pan G, Ma Q, Zhang J, Pei D. 2015. The genetic diversity and introgression of *Juglans regia* and *Juglans sigillata* in Tibet as revealed by SSR markers. Tree Genet Genomes. 11(1):1.

[CIT0030] Wang M, Zhang C, Li M, Gao X. 2019. The complete chloroplast genome sequence of *Rosa banksiae* var. normalis (Rosaceae). Mitochondrial DNA Part B. 4(1):969–970. 10.1080/23802359.2019.1674200 PMC7707283 33366083

[CIT0031] Wang Q, Hu H, An J, Bai G, Ren Q, Liu J. 2018. Complete chloroplast genome sequence of *Rosa roxburghii* and its phylogenetic analysis. Mitochondrial DNA B Resour. 3(1):149–150. 33490491 10.1080/23802359.2018.1431074 PMC7800368

[CIT0032] Wang SQ, Chen JF, Zhu ZM. 2020. The complete chloroplast genome sequence of *Rosa filipes* (Rosaceae). Mitochondrial DNA Part B. 5(2):1376–1377.

[CIT0033] Xue S, Shi T, Luo W, Ni X, Iqbal S, Ni Z, Huang X, Yao D, Shen Z, Gao Z. 2019. Comparative analysis of the complete chloroplast genome among *Prunus mume*, *P. armeniaca*, and *P. salicina*. Hortic Res. 6:89. 31666958 10.1038/s41438-019-0171-1 PMC6804877

[CIT0034] Yin X, Liao B, Guo S, Liang C, Pei J, Xu J, Chen S. 2020. The chloroplasts genomic analyses of *Rosa laevigata*, *R. rugosa* and *R. canina*. Chin Med. 15(1):1–11. 32082412 10.1186/s13020-020-0298-x PMC7020376

[CIT0035] Zhang SD, Zhang C, Ling LZ. 2019. The complete chloroplast genome of *Rosa berberifolia*. Mitochondrial DNA Part B. 4(1):1741–1742.

[CIT0036] Zhao X, Gao C. 2020. The complete chloroplast genome sequence of *Rosa minutifolia*. Mitochondrial DNA B Resour. 5(3):3320–3321. 33458151 10.1080/23802359.2020.1817807 PMC7782609

