# Supplementary figures and images for: The first chloroplast sequence of Rosa davurica Pall. var. Davurica
Source: Mitochondrial DNA B Resour. 2023 Jun 13;8(6):668–72. doi: 10.1080/23802359.2023.2220431 (PMC10266123; doi:10.1080/23802359.2023.2220431)

# Trans-splicing Genes

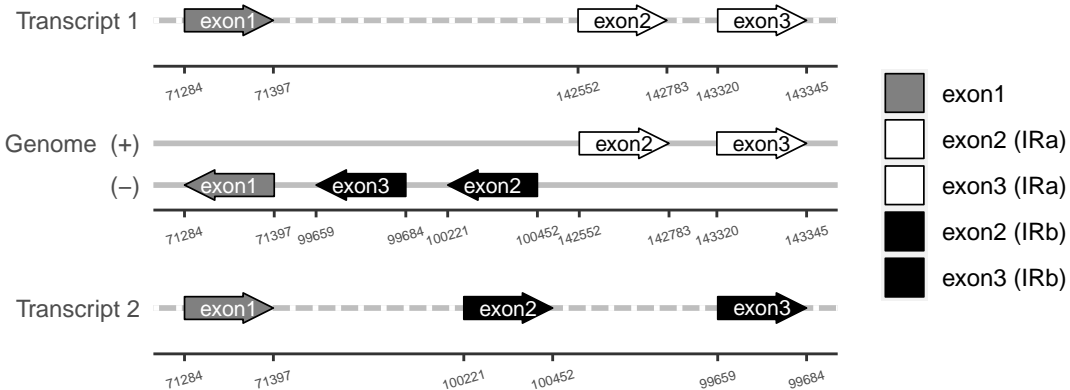

Supplement: Supplemental Material [file TMDN_A_2220431_SM5133.pdf]

# Cis-splicing Genes

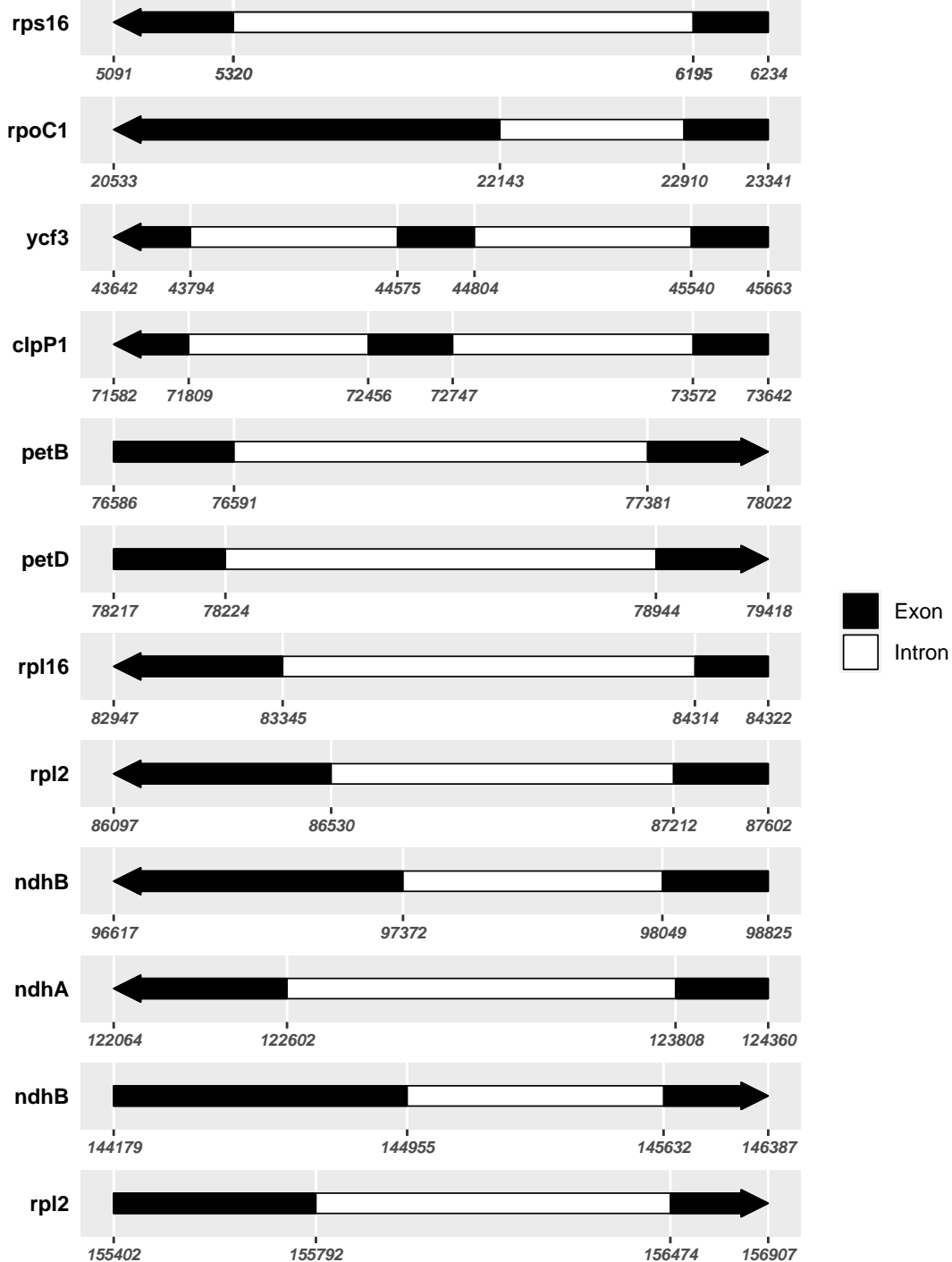

Supplement: Supplemental Material [file TMDN_A_2220431_SM5132.pdf]

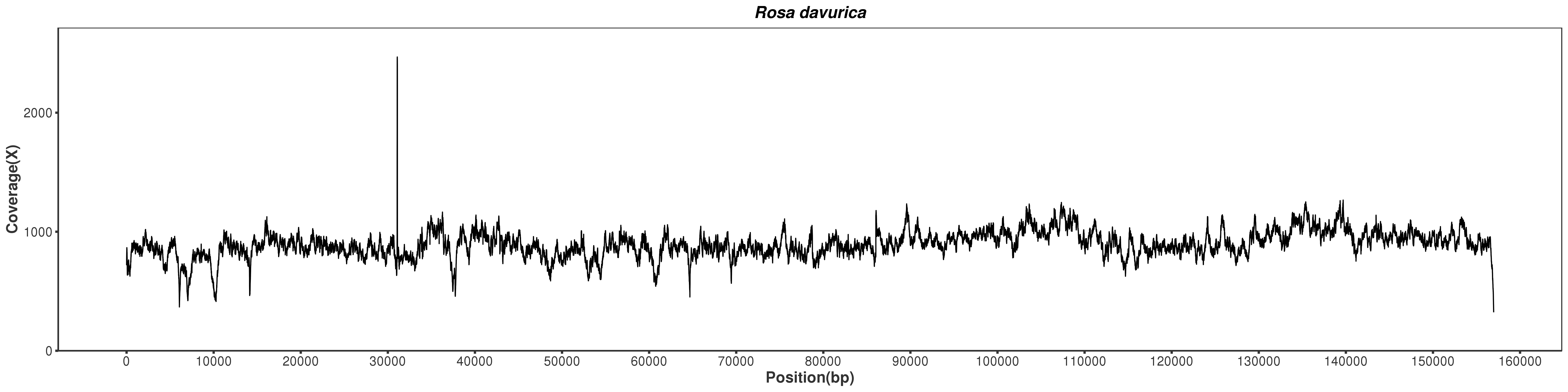

Supplement: Supplemental Material [file TMDN_A_2220431_SM5130.png]
